# Assessing how routes to diagnosis vary by the age of patients with cancer: a nationwide register-based cohort study in Denmark

**DOI:** 10.1186/s12885-022-09937-y

**Published:** 2022-08-19

**Authors:** B. Danckert, N. L. Christensen, A. Z. Falborg, H. Frederiksen, G. Lyratzopoulos, S. McPhail, A. F. Pedersen, J. Ryg, L. A. Thomsen, P. Vedsted, H. Jensen

**Affiliations:** 1 The Danish Cancer Society Research Center, Copenhagen, Denmark; 2 Department of Respiratory Diseases and Allergy, Aarhus University Hospital, Aarhus, Denmark; 3 Research Unit for General Practice, Aarhus, Denmark; 4 Haematological Research Unit, Department of Haematology, Odense University Hospital, Odense, Denmark; 5 Department of Clinical Research, University of Southern Denmark, Odense, Denmark; 6 Academy of Geriatric Cancer Research (AgeCare), Odense University Hospital, Odense, Denmark; 7 Epidemiology of Cancer Healthcare and Outcomes (ECHO) Research Group, Department of Behavioural Science and Health, University College London, London, UK; 8 National Disease Registration Service, NHS Digital, Leeds, UK; 9 Research Unit of Geriatric Medicine, Department of Geriatric Medicine, Odense University Hospital, Odense, Denmark

**Keywords:** Denmark, Neoplasm (MeSH), Cancer, “Early Detection of Cancer” (MeSH)

## Abstract

**Background:**

Older patients with cancer have poorer prognosis compared to younger patients. Moreover, prognosis is related to how cancer is identified, and where in the healthcare system patients present, i.e. routes to diagnosis (RtD). We investigated whether RtD varied by patients’ age.

**Methods:**

This population-based national cohort study used Danish registry data. Patients were categorized into age groups and eight mutually exclusive RtD. We employed multinomial logistic regressions adjusted for sex, region, diagnosis year, cohabitation, education, income, immigration status and comorbidities. Screened and non-screened patients were analysed separately.

**Results:**

The study included 137,876 patients. Both younger and older patients with cancer were less likely to get diagnosed after a cancer patient pathways referral from primary care physician compared to middle-aged patients. Older patients were more likely to get diagnosed via unplanned admission, death certificate only, and outpatient admission compared to younger patients. The patterns were similar across comorbidity levels.

**Conclusions:**

RtD varied by age groups, and middle-aged patients were the most likely to get diagnosed after cancer patient pathways with referral from primary care. Emphasis should be put on raising clinicians’ awareness of cancer being the underlying cause of symptoms in both younger patients and in older patients.

**Supplementary Information:**

The online version contains supplementary material available at 10.1186/s12885-022-09937-y.

## Introduction

With an ageing population, older patients constitute a larger proportion of patients with cancer across the western societies [1–3]. At the same time, older patients with cancer have poorer prognosis compared to younger patients [3]. The prognostic deficit seen in older patients with cancer is associated with many factors such as higher levels of comorbidity and frailty, and less evidence-based treatment (as old patients are often excluded from clinical trials). Yet, the reasons for the poorer prognosis among older patients with cancer are not fully understood.

Research reports that prognosis is also associated with routes to diagnosis (RtD) [4–6], which is often defined as the likely series of key interactions between patients and the healthcare system during the course of disease from presentation until cancer diagnosis [4, 7]. It is also reasonable to expect that RtD may be affected by factors that relate to patients’ age [3]. For instance, many cancer screening programs are not available after a certain age. Likewise, the presence of comorbidities, which are considerably more common among older people, may cause patients to have multiple contacts with the healthcare system – including more acute contacts such as an unplanned hospital admission – during which a cancer may be suspected or identified. Indeed, research indicates that older patients are more likely to get diagnosed via such emergency route [4, 6, 8]. However, research is sparse, and stems mainly from England. It is unknown whether the results can be replicated in other healthcare systems.

Since the turn of the millennium, several countries have established programs intended to ensure faster and earlier diagnosis of cancer as a mean to improve prognosis and survival [9–12]. An example is the standardised cancer patient pathways (CPPs) introduced in Denmark in 2008 and later in Sweden and Norway. The CPPs define organizational procedures as well as a schedule with recommended time-frames for clinical interventions from referral to treatment [12–14]. While a few CPPs have age restrictions, the programs should otherwise be available irrespective of age. Nevertheless, a recent study from Norway indicates that a lower proportion of older patients with cancer underwent a CPP. However, the Norwegian study did not account for other RtD, such as unplanned admissions/emergencies, and was based on data from the years immediately after the introduction of CPPs in Norway [15].

The aim of this study was to investigate the association between age and RtD among all patients with cancer diagnosed in Denmark from 2014 to 2017 by linking and analysing routinely collected Danish register data. We analysed cancers with a national screening program and cancers not detected by a national screening program (i.e. symptomatic cancers) separately because they differ fundamentally with regard to the primary contact to the healthcare system: For screening, patients are typically asymptomatic, and the diagnostic process is initiated by the health authorities through an invitation; for all other routes, patients present with symptoms or signs (although the cancer may be diagnosed upon presentation with symptoms or signs for other diseases than cancer) [10, 16, 17].

We hypothesized that the proportion of cancers diagnosed upon screening for each age group corresponded to the degree of participation in screening programs. We also hypothesized that among symptomatic cancer patients, the older patients were less likely to get diagnosed with cancer following a referral to a CPP from primary care than younger patients.

## Materials and methods

The study was designed as a population-based national cohort study using routinely collected Danish registry data. The study’s data sources, population, and designation of RtD have been described in details elsewhere [18], and are only summarized here.

### Settings

The study was based in Denmark, which has 5.8 million inhabitants. The Danish health care system is tax-based and offers free and equal access to most medical services. More than 98% of citizens are registered at a general practitioner (GP), who act as a gate-keeper to the secondary health care system, except to emergency ward, eye specialists and ear-nose and throat specialists. Since 2009 more than 30 Cancer patient pathways (CPPs) covering approximately 40 cancer diseases have been introduced [12]. National screening programmes exist for breast cancer (women aged 50–64 years), cervical cancer (women aged 23–69), and colorectal cancer (both sexes aged 50–74 years) [12, 19]. All of these screening programmes were fully implemented throughout the study period (Additional file, section 1).

### Data sources

The study included data from a range of Danish nation-wide population registries and clinical databases considered to have high completeness and validity [20]; e.g. The Danish Cancer Registry includes 90–97% of all solid tumours [21–23]. From each register, we used the following information. The Danish Cancer Registry [24]: cancer diagnosis, date of diagnosis, region of residence, age, and sex; the National Patient Registry [25]: data on all contacts with somatic hospitals in Denmark including information on inpatient and outpatient visits including diagnoses, dates, department codes, and CPPs; the Danish Breast Cancer Group’s database [26]: information regarding breast cancer screening; the Danish Colorectal Cancer Database [27]: information regarding screening for rectum and colon cancer; the Danish Quality Database for Cervical Cancer Screening [28]: information regarding screening for cervical cancer; and the cause of death registry [29]: data on date of death. From Statistics Denmark we linked information regarding income, educational level, marriage, immigration status, and cohabitation.

Data from the registries and databases were linked at the personal level using a pseudomised version of the unique personal registration number assigned to all citizens in Denmark at birth or immigration.

### Study population

We included all patients registered in the *Danish Cancer Registry* with an invasive cancer excluding non-melanoma skin cancer (ICD-10: C00-C43 & C45-C97) aged 18 years or more at time of diagnosis within the period of 1 January 2014 to 31 December 2017. Both patients diagnosed with one or multiple cancer diagnoses in the study period were included implying that a person may appear more than once in the data set with different tumours. Males diagnosed with breast cancer were excluded, as no designated diagnostic pathway for this patient group exists in Denmark.

### Defining outcome: routes to diagnosis (RtD)

RtD was the outcome of the study. Inspired by Elliss-Brookes et al. [4], we defined the concept as the series of interactions between the patient and the healthcare system that most likely lead to cancer diagnosis, based on how the patient was referred into secondary care. The categorisation of RtD was based on cancer registrations for all identified patients in the Danish Cancer Registry linked to data on all hospital contacts from the National Patient Registry as well as data from the clinical databases regarding screening for breast cancer, colorectal cancer, and cervical cancer.

Parallel to previous research [18], we defined eight mutually exclusive RtD:

1. **Death certificate only (DCO)**: The patient was registered as diagnosed by a death certificate only in the Danish Cancer Registry.
2. **Screening**: The patient was registered as detected through a national screening program and diagnosed with breast cancer, colon cancer, rectal cancer, or cervical cancer. For cervical cancer, screen detected implied being registered with a positive smear test up to three months before diagnosis.
3. **CPP – primary care**: The patient was referred to a CPP (including the CPP concerning “non-specific signs and symptoms that could be cancer” (NSSC-CPP)) by a primary care healthcare professional (i.e. any medical doctor working in primary care regardless of medical specialty).
4. **CPP – secondary care**: The patient was referred to a CPP (including the CPP concerning “non-specific signs and symptoms that could be cancer” (NSSC-CPP)) by other than a primary care healthcare professional such as a medical specialist in a hospital.
5. **Unplanned admission**: The patient had an inpatient hospital admission coded as acute within 30 days before the cancer diagnosis and no start of a CPP prior to this.
6. **Planned admission for other reasons than cancer**: The patient had an admission coded as a planned admission within 30 days before the cancer diagnosis, and no start of a CPP or an unplanned admission prior to this.
7. **Outpatient**: The patient had an outpatient visit (outpatient hospital specialist clinic) recorded within 30 days before the cancer diagnosis, and no start of a CPP, unplanned admission, or planned admission prior to this.
8. **Unknown**: All others.

We designated a specific RtD for each cancer by first defining DCO and screened cases, second categorizing the remaining cases according to the earliest route registered. In cases with multiple routes registered on the same day, we designated the route ranking highest in the order outlined above.

### Defining age at the time of diagnosis

The main exposure of interest was patients’ age at the time of diagnosis as registered in the Danish Cancer Registry. Age was categorized into seven categories: 18–39, 40–49, 50–59, 60–69, 70–79, 80–89, and 90+ years.

### Defining other variables

Cancer diagnoses were categorized into 23 specific groups of diagnosis based on the ICD-10 diagnosis code (see Additional file section 2). Patients level of comorbidities was measured using the Charlson Comorbidity Index [30], based on registrations in *the National Patient Registry* ten years before the cancer diagnosis, and divided into three levels: low (CCI-score = 0), medium (CCI-score = 1–2), and high (CCI-score > 2). Region of residence referred to the five administrative health care regions in Denmark, which are responsible for all cancer diagnostic procedures and cancer treatment. Cohabitating/being married was defined as a patient being married, living together with another person, with whom he/she had children, or living together with a person of the opposite sex and a maximum of 15 years of age difference [31]. Income was defined as disposable personal income excluding taxes and interest expenses (depreciated to 2015-value) and afterwards divided into quartiles. Educational attainment was categorized according to International Standard Classification of Education (ISCED) into categories “short” (ISCED levels 1–2), “medium “(ISCED levels 3-4), “long” (ISCED levels 5–8), and “unknown”. Immigration status was measured using a variable taking on the value 1 for immigrants and descendants defined as individuals for whom none of the parents were Danish citizens nor born in Denmark, in accordance with Statistics Denmark.

### Statistical analyses

We performed multinomial logistic regression to investigate the association between age and RtD, and estimated probabilities of starting the diagnostic pathway through each of the RtDs using marginal predicted probability with 95% confidence intervals (95% CI) for all age groups. Marginal predicted probabilities were computed with covariates at their observed value. The multinomial logistic regressions were performed both case-mix adjusted (sex, diagnosis, region of residence, and year of diagnosis), additionally adjusted for comorbidities and fully adjusted (adding income, education, immigration status and cohabitation).

Our primary focus was on symptomatic patients (i.e. patients *not* diagnosed through the screening route) because these are diagnosed through pathways where healthcare professionals could choose to take action based on symptoms and signs. Therefore, these pathways are clinically modifiable. Screening, on the other hand, constitutes a route intended to identify cancer among asymptomatic patients and is restricted by age. Thus, we analysed age in relation to patients diagnosed through the screening route and patients diagnosed outside screening route separately. For the analysis concerning the screening route, logistic regression rather than multinomial logistic regression was performed because of the binary outcome, i.e. screening route vs. non-screening route.

For patients not diagnosed through the screening route, we performed sensitivity analyses of the following interactions in the multinomial logistic regression: Age and sex, age and socio-economic position (age*income, age*education), and age and health status (age*multimorbidity). We also ran analyses in which we excluded diagnosis for which the CPP referral guidelines included an age restriction implying that only patients above a certain age threshold were referred to CPPs when displaying specific symptoms; for instance, the referral guidelines for the CPP for colorectal cancer specifies that patients have to be older than 40 years. Diagnoses with CPP age restrictions included cancer in colon, rectum, urinary tract, lung, oesophagus, stomach, larynx, and hypopharynx.

All analyses were undertaken at the tumour level, implying that multiple tumours were included in the analyses as independent observations. All statistical procedures were performed using Stata 16.

## Results

The Danish Cancer Registry contained records of 185,339 cancers identified within the study period. Following the inclusion criteria, patients with missing information regarding sex (*n* = 786), younger than 18 years (*n* = 931), with benign cancer or unknown location (*n* = 21,070), dysplasia (*n* = 16,881) or mola (*n* = 377), and males with breast cancer (*n* = 159) were excluded. Furthermore, 480 observations from 468 individuals were deleted because of missing information regarding region of residence, and 404 observations (from 398 patients) were excluded due to missing information regarding cohabitation, income, or immigration status. Finally, we excluded 839 cancers registered as detected by the national screening programs, because the patients were outside the age range outlined in the national guidelines. The final study sample consisted of 137,876 patients with a total of 143,389 cancers.

Of all cancers, 69,888 (48.7%) were diagnosed among women and 73,501 (51.3%) among men. 59.7% of cancers were diagnosed among patients aged 60 to 79 (Table 1). A total of 9932 cancers (6.9%) were identified via a screening route. Table 1 displays the distribution of covariates; in section 3 of the Additional file the distribution of covariates is displayed by RtD.

**Table 1:** Characteristics of the included patients with cancer stratified by sex (and total)

	Women		Men		Total	
	*n*	(%)	*n*	(%)	*n*	(%)
**Total**	69,888	(48.7)	73,501	(51.3)	143,389	(100.0)
**Age (mean, sd)**	66.1	(14.1)	68.1	(11.9)	67.2	(13.1)
**Age groups**						
18–39	3148	(4.5)	2005	(2.7)	5153	(3.6)
40–49	5763	(8.2)	2845	(3.9)	8608	(6.0)
50–59	11,315	(16.2)	9398	(12.8)	20,713	(14.4)
60–69	18,682	(26.7)	22,884	(31.1)	41,566	(29.0)
70–79	18,995	(27.2)	25,042	(34.1)	44,037	(30.7)
80–89	10,229	(14.6)	10,172	(13.8)	20,401	(14.2)
≥ 90	1756	(2.5)	1155	(1.6)	2911	(2.0)
**Routes to Diagnosis**						
Death certificate only	318	(0.5)	312	(0.4)	630	(0.4)
Screening	7597	(10.9)	2335	(3.2)	9932	(6.9)
CPP primary sector referral	30,032	(43.0)	36,227	(49.3)	66,259	(46.2)
CPP secondary sector referral	15,192	(21.7)	13,651	(18.6)	28,843	(20.1)
Unplanned admission	10,491	(15.0)	12,336	(16.8)	22,827	(15.9)
Planned admission	681	(1.0)	814	(1.1)	1495	(1.0)
Outpatient visit	3560	(5.1)	5429	(7.4)	8989	(6.3)
Unknown route	2017	(2.9)	2397	(3.3)	4414	(3.1)
**Year of diagnosis**						
2014	17,314	(24.8)	18,268	(24.9)	35,582	(24.8)
2015	17,503	(25.0)	18,319	(24.9)	35,822	(25.0)
2016	17,467	(25.0)	18,390	(25.0)	35,857	(25.0)
2017	17,604	(25.2)	18,524	(25.2)	36,128	(25.2)
**Region**						
Northern Denmark	7397	(10.6)	8156	(11.1)	15,553	(10.8)
Central Denmark	14,667	(21.0)	16,489	(22.4)	31,156	(21.7)
Southern Denmark	15,911	(22.8)	17,026	(23.2)	32,937	(23.0)
Capital	20,441	(29.2)	19,405	(26.4)	39,846	(27.8)
Zealand	11,472	(16.4)	12,425	(16.9)	23,897	(16.7)
**Comorbidity (CCI)**						
None	39,502	(56.5)	35,706	(48.6)	75,208	(52.5)
Moderate (CCI = 1–2)	20,880	(29.9)	23,699	(32.2)	44,579	(31.1)
High (CCI > 2)	9506	(13.6)	14,096	(19.2)	23,602	(16.5)
**Diagnosis group**						
Head & neck	1191	(1.7)	2708	(3.7)	3899	(2.7)
Oesophagus	590	(0.8)	1559	(2.1)	2149	(1.5)
Stomach	855	(1.2)	1662	(2.3)	2517	(1.8)
Colon	7086	(10.1)	7451	(10.1)	14,537	(10.1)
Rectum	2595	(3.7)	4100	(5.6)	6695	(4.7)
Liver	515	(0.7)	1262	(1.7)	1777	(1.2)
Pancreas	1895	(2.7)	2052	(2.8)	3947	(2.8)
Lung	9371	(13.4)	9513	(12.9)	18,884	(13.2)
Melanoma	5167	(7.4)	4570	(6.2)	9737	(6.8)
Breast	19,128	(27.4)	0	()	19,128	(13.3)
Uterus	3281	(4.7)	0	()	3281	(2.3)
Ovary	2128	(3.0)	0	()	2128	(1.5)
Female genitals	2018	(2.9)	0	()	2018	(1.4)
Prostate	0	()	18,134	(24.7)	18,134	(12.6)
Male Genitals	0	()	1457	(2.0)	1457	(1.0)
Kidney	1470	(2.1)	2705	(3.7)	4175	(2.9)
Bladder	974	(1.4)	2720	(3.7)	3694	(2.6)
Eye, Brain, CNS	1037	(1.5)	1360	(1.9)	2397	(1.7)
Endocrine glands	1114	(1.6)	468	(0.6)	1582	(1.1)
Lymphoma	2328	(3.3)	2933	(4.0)	5261	(3.7)
Multiple myeloma	840	(1.2)	1092	(1.5)	1932	(1.3)
Leukaemia	1499	(2.1)	2268	(3.1)	3767	(2.6)
Other	4806	(6.9)	5487	(7.5)	10,293	(7.2)
**Educational level**						
Low	26,210	(37.5)	22,100	(30.1)	48,310	(33.7)
Medium	25,134	(36.0)	33,321	(45.3)	58,455	(40.8)
High	16,865	(24.1)	16,328	(22.2)	33,193	(23.1)
Missing/unknown	1679	(2.4)	1752	(2.4)	3431	(2.4)
**Disposable income (quartiles)**						
1st	19,019	(27.2)	16,123	(21.9)	35,142	(24.5)
2nd	18,936	(27.1)	18,361	(25.0)	37,297	(26.0)
3rd	17,551	(25.1)	17,499	(23.8)	35,050	(24.4)
4th	14,382	(20.6)	21,518	(29.3)	35,900	(25.0)
**Marital status**						
Single	31,222	(44.7)	21,966	(29.9)	53,188	(37.1)
Married/co-habiting	38,666	(55.3)	51,535	(70.1)	90,201	(62.9)
**Immigration status**						
Not immigrant/descendent	66,079	(94.5)	70,002	(95.2)	136,081	(94.9)
Immigrant/descendent	3809	(5.5)	3499	(4.8)	7308	(5.1)

Note: percentages may not total up to 100% due to rounding

The mean age was lowest among patients diagnosed through the screening route (61.3 years) and highest among patients diagnosed via DCO (79.6 years). For the remaining routes, the mean age was 66.8 years for CPP with referral from primary care, 66.6 for CPP with referral from secondary care, and 67.2 for the outpatient route. For planned admissions, the mean age was 65.1, and for the unknown route and unplanned admissions it was 68.2 and 71.0, respectively (Additional file, section 3).

### Age and symptomatic RtD

**Fig. 1:**
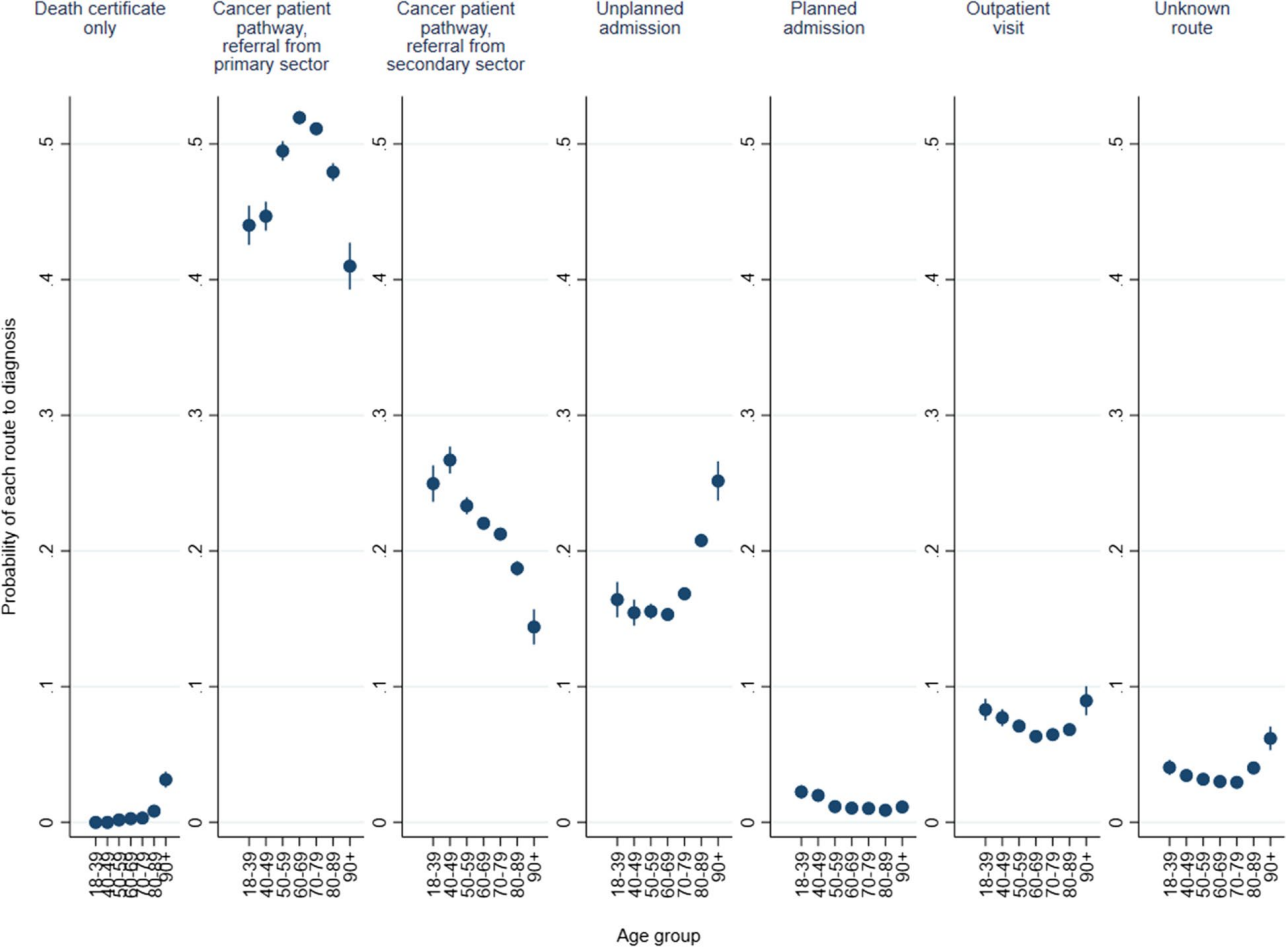
Marginal probability of a cancer diagnosis via each route to diagnosis by age groups based on the fully adjusted multinomial regression model (covariates include sex, year of diagnosis, region, diagnosis group, comorbidities, education, income, marital status, and immigration status). Patients diagnosed through the screening route were excluded

The probability of being diagnosed through each RtD varied across age groups (Fig. 1). While CPP with referral from primary care was the most likely RtD across all age groups, it was more likely among patients aged 50–59, 60–69, and 70–79 compared to other age groups; the probability being 49.5% (CI = 48.8–50.2), 51.9% (CI = 51.5–52.4), and 51.1% (CI = 50.7–51.6), respectively. For older patients with cancer, the probability of being diagnosed after CPP referral from primary care was 47.9% (CI = 47.3–48.6) for patients aged 80–89 and 41.0% (CI = 39.3–42.7) for patients aged 90 or more. For the younger patients, the probability was 44.0% (CI = 42.6–45.5) for patients aged 18–39 and 44.7% (CI = 43.6–45.8) for patients aged 40–49 (see Fig. 1).

Older patients with cancer were more likely to get diagnosed via unplanned admission, and for the oldest age group, also via death certificate only, compared to younger patients. The youngest age groups were overrepresented with regard to CPP with referral from hospital and planned admissions other than CPPs. They also displayed greater likelihood of being diagnosed upon outpatient visits compared to patients aged 60–89 (Fig. 1).

The associations between age and RtD were substantially similar irrespective of whether the analyses were case-mix adjusted, additionally adjusted for comorbidities or fully adjusted though the age discrepancies were slightly reduced when including (A) comorbidities and (B) comorbidities and socioeconomic factors in the regression model (Additional file section 4 and 5).

In subsequent analyses, we have investigated the pattern across age groups across the four main cancer types (breast, colon, lung, and prostate) (Additional file section 6). Overall, the pattern remained, though for breast cancer, the likelihood of CPP with referral from primary care was highest among the elderly, which is likely a consequence of the breast cancer screening programme covering women aged 50–64 years).

**Fig. 2:**
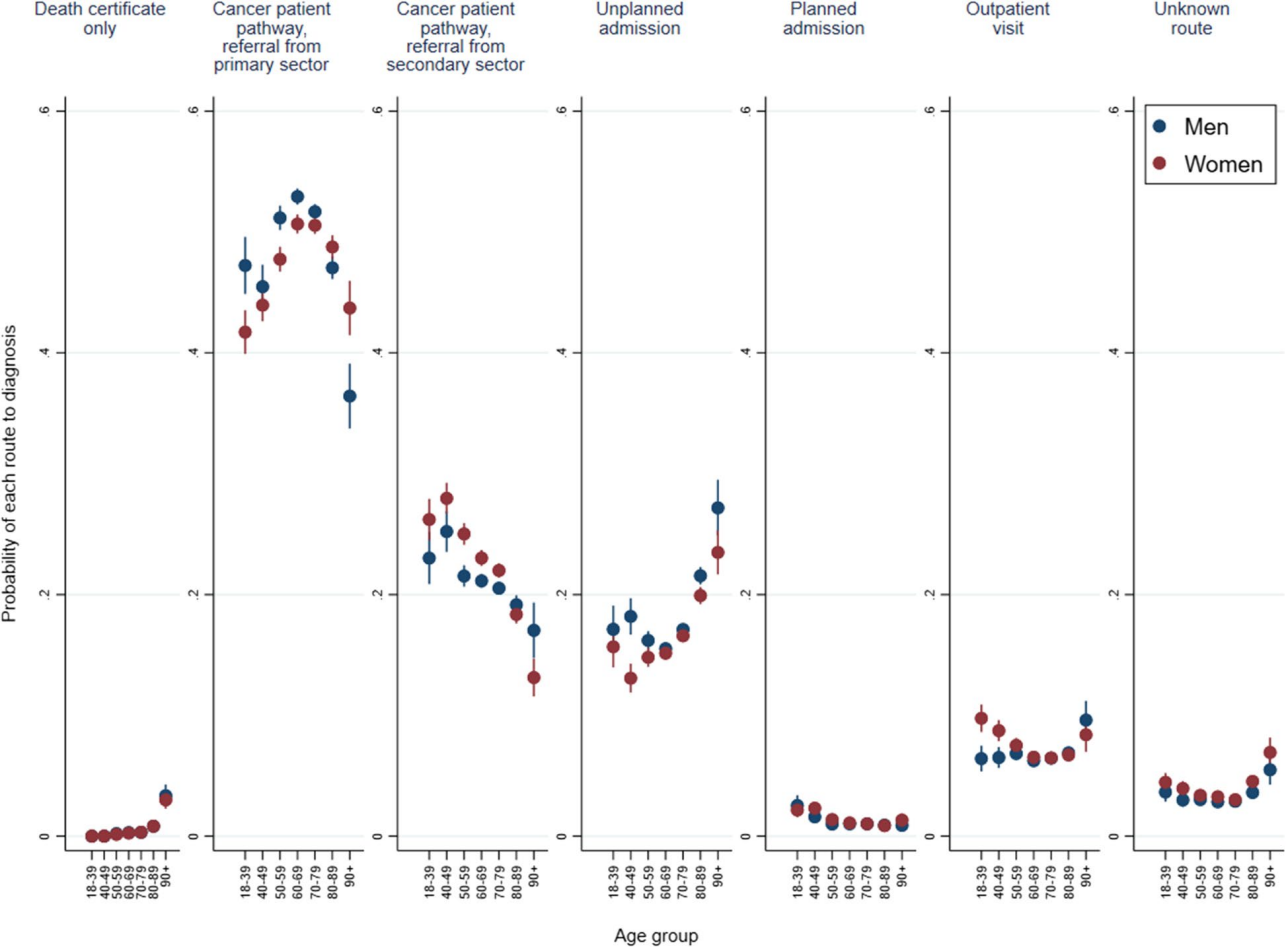
Marginal probability of a cancer diagnosis via each routes to diagnosis by age groups and sex based on the fully adjusted multinomial regression model (covariates include year of diagnosis, region, diagnosis group, comorbidities, education, income, marital status and immigration status). Patients diagnosed through the screening route were excluded

Sensitivity analyses showed that the association between age and RtD was broadly similar for each sex. Yet, younger men were slightly more likely to get diagnosed after CPP referral from primary care, but less likely to get diagnosed after a CPP referral from secondary care and via an outpatient route compared to younger women. In contrast, older men were less likely to get diagnosed after CPP referral from primary care than older women were, but more likely than older women to get diagnosed after CPP referral from secondary care or by an unplanned admission (Fig. 2).

In additional sensitivity analyses, we excluded diagnoses for which the CPP referral guidelines included an age restriction (i.e. cancers of colon, rectum, urinary tract, oesophagus, stomach, lung, larynx and hypopharynx). The results of these subsequent analyses are displayed in Additional file section 7. The overall pattern across age groups remained the same, though the likelihood of e.g. CPP with referral from primary care was slightly higher across all age groups for the diagnoses without age restrictions, and the age difference in likelihood of CPP with referral from primary care was somewhat smaller.

We also tested whether the association between age and RtD varied by (a) socio-economic position (income and educational level) and (b) health status (the comorbidity measure) for the symptomatic RtD. Neither the interaction between age and income nor the interaction between age and education showed any consistent significant pattern. With regard to comorbidities, the patterns were largely similar across patients with none, moderate or high levels of comorbidities; while we did see some significant variation depending on the level of comorbidities, this did not substantially alter the general relationship between RtD and age. Most notably, while the probability of getting diagnosed based on DCO was higher among the oldest age groups for all levels of comorbidities, this was particularly so among patients with moderate or high levels of comorbidities (Additional file section 8).

### Age and screening RtD

**Fig. 3:**
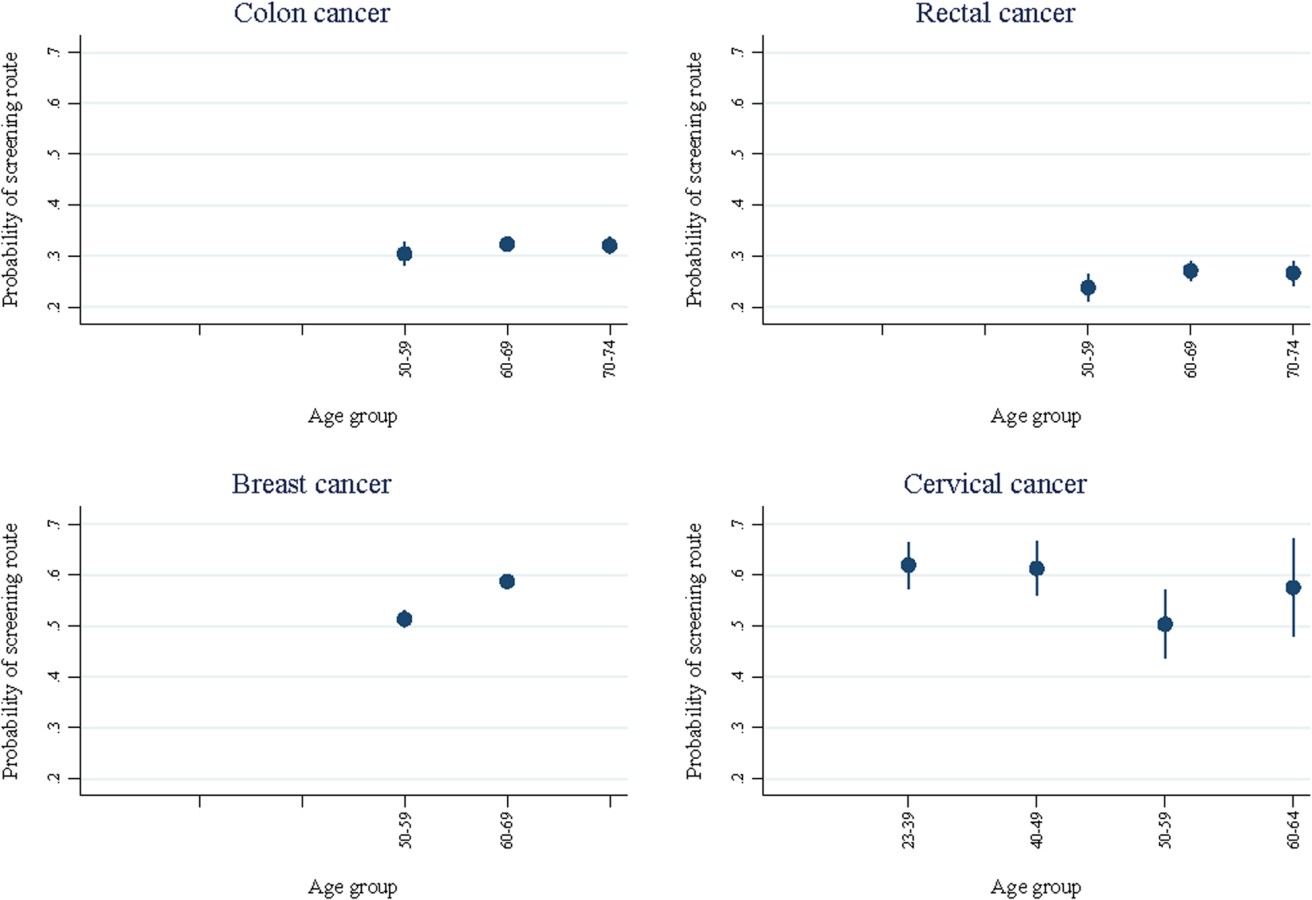
Marginal probability of screening by age groups for cancer sites with a national screening programme based on the fully adjusted multinomial regression model (covariates include sex, year of diagnosis, region, comorbidities, education, income, marital status and immigration status)

Figure 3 shows the probability of being diagnosed following a screening route depending on patients’ age for each of the four cancers – colon, rectum, breast, and cervical cancer (Additional file section 9).

For each of these cancer diagnoses, the screening route covered a proportion of the specific cancers within the entire age range for which screening was offered, ranging from 26.2% in rectum cancer to 59.1% in cervical cancer. For patients with breast cancer aged 60–69, screening detected 58.8% (CI = 57.5–60.1), and the equivalent number for patients with cervical cancer aged 18–39 and 40–49 years was 62.0% (CI = 57.4–66.5) and 61.3% (CI = 56.1–66.5), respectively.

The probability of being diagnosed with cervical cancer through screening was approximately 10 percentage points lower among patients aged 50–59 compared to the youngest age groups and compared to patients aged 60–64; the probability being 62.0% (CI = 57.4–66.5) and 61.3% (CI = 56.1–66.5) in patients aged 18–39 and 40–49, respectively, and 50.3% (43.6–57.1) and 57.5% (CI = 48.0–67.1) in patients aged 50–59 and 60–64 years, respectively. For breast cancer, the probability of being diagnosed through screening was 7 percentage points higher among patients aged 60–69 compared to patients aged 50–59 (58.8% (CI = 57.5–60.1) compared to 51.4% (CI = 49.8–52.9)). For patients diagnosed with colon cancer, the probability of being diagnosed via screening was close to 30% for all age groups (30.4% (CI = 28.1–32.8), 32.3% (CI = 30.9–33.7) and 32.0% (CI = 30.4–33.7) for age group 50–59, 60–69 and 70–74, respectively). Finally, the probability of being diagnosed with rectal cancer was 23.8% (CI = 21.1–26.5) among patients aged 50–59, and 27.1% (CI = 25.3–29.0) and 26.7% (CI = 24.2–29.1) for patients aged 60–69 and 70–74, respectively.

The associations between age and screening were substantially similar irrespective of whether the analyses were case-mix adjusted or fully adjusted (Additional file section 9 and 10).

## Discussion

This register-based nationwide study included more than 140,000 cancers and demonstrated that the probability of getting diagnosed through a specific RtD varied by the age of the patients (in both case-mix adjusted analyses and analyses adjusting for socio-economic characteristics and comorbidity). Among patients diagnosed with cancer outside screening programs, our study showed that patients aged 80 years or older were more likely to get diagnosed by DCO or by an unplanned admission, and less likely to get diagnosed after a CPP referral from primary care compared to patients aged 50–79 years. The pattern was even more profound for patients aged 90 years or more. Patients below 50 years were also less likely to get diagnosed after a CPP referral from primary care, yet more likely to be diagnosed after CPP referral from secondary care compared to patients aged 50–79 years.

Among screen-detected cancers, the probability of having a screen-detected cervical cancer was higher among the youngest age groups (aged 23–49) compared to patients aged 50–59, while for patients with breast cancer the probability of being diagnosed after screening was higher among patients aged 60–69 than among patients aged 50–59.

### Comparison with other literature

Despite this being the first large scale study to report the associations between age and RtD in a Nordic healthcare system, our findings still bear similarities with findings from other countries and smaller studies [4, 6, 32–34].

In parallel with findings from England, while CPP with referral from primary care was the most frequent RtD across all age groups, the oldest patients were less likely to get diagnosed via this route compared to younger age groups [4, 6]. A smaller study from Denmark contradicts our findings of older patients being less likely to get diagnosed after CPP referral from primary care, as Jensen and colleagues reported no age differences [32]. However, this discrepancy may be due to selection and information bias in the former study, as the study was based on questionnaire data [32].

A study from Norway showed that older patients were less likely to complete a CPP than younger patients [15]. Although the Norwegian study did not account for other RtD, the findings in conjunction with ours indicates that older patients are less likely to get diagnosed after CPP referral from primary care – especially as the organization of CPPs in Norway is similar to the Danish [14, 15].

Elliss-Brookes et al. also reported that older patients constituted a larger proportion of patients diagnosed by DCO or after an emergency admission compared to younger patients with cancer in England [4]. This is in line with our findings. Especially emergency presentation, which relates to unplanned admission investigated in our study, has been shown to be associated with older age [6, 34].

Our finding of the youngest patients being less likely to get diagnosed after CPP referral from primary care contrasts the reporting from both England [4] and a smaller Danish survey [32]. In England, the proportion of younger patients diagnosed by two-week-wait referral was similar or slightly higher than the proportion of patients from 50 to 69 years of age [4]. The reasons for this discrepancy is unknown, whereas the discrepancy with the Danish survey study may be due to the higher number of young patients in the present study [32].

Interestingly, screening programs detected a large share of the cervix cancers among the youngest women, even though the participation rates in the national screening program in Denmark is lower within this age group compared to older age groups [35]. Participation rates in breast cancer screening are fairly even across the invited age groups while we find that screening detected a slightly higher share of patients aged 60–69 compared to 50–59 [36, 37].

### Methodological considerations

Major strengths of the study are the high quality of data that cover the entire Danish cancer population, and that Danish national registers are reliable and have a high degree of completeness [20]. We facilitated the analyses by excluding relatively few observations, but this is unlikely to substantially to have impacted the results. Arguably, using registry data, registration errors should be acknowledge, but such registration errors are unlikely to systematically bias the results. Also, our methods were robust, as the results were similar in sensitivity analyses.

Some limitations, however, also relates to the data. The RtDs were defined in line with most related studies in the field by using a contextual definition, in contrast to a clinical definition, which relates to the patient’s medical condition [7]. Despite, the definition being contextual, increased adverse prognosis among unplanned admissions supports the use of a contextual definition as a marker of clinical severity [4, 7, 18]. An additional limitation is that the registers do not contain data that allow adjustment for important covariates such as body mass index, smoking or alcohol consumption.

### Interpretation and implications

Patients with cancer aged 50–79 years were most likely to get diagnosed after CPP referral from primary care in symptomatic patients. Along with screening, this RtD may, from a prognostic view, be seen as the most optimal RtD for a given patient [38], as these two RtDs are associated with the best prognosis and highest level of patient satisfaction [4, 6, 33, 39].

Despite symptomatic patients aged 50–79 years were most likely to get diagnosed after CPP referral from primary care, more than four out of ten symptomatic patients with cancer in this age range were still diagnosed via an RtD associated with a worse prognosis. This emphasizes that there may be room for improvement in the diagnosis of cancer among mid-aged patients.

We found that patients younger than 50 years were less likely to get diagnosed after referral from primary care to a CPP. General practitioners not suspecting cancer as the cause of young patients’ symptoms may explain this [32, 40]. Yet, patients with cancer aged 50 years or younger comprise 16% of all patients with cancer in Denmark [41], indicating that cancer diagnoses could be missed initially in this age group. Thus, although relatively uncommon in patients below 50 years, cancer remains a potential differential diagnosis whenever a patient presents to healthcare, now or in the future, as the incidence of cancer in patients aged 20–50 years are increasing [41].

Despite patients aged 70 years or older constitute almost half of all patients with cancer in Denmark [42], our study shows that the oldest patients are less likely to get diagnosed after CPP referral from primary care. This may be related to the higher prevalence of comorbidity in older patients for two reasons: The existence of other morbidities may mislead clinicians to contribute signs and symptoms to the already existing morbidity rather than an underlying cancer [43]. Consequently, the patients may not be referred to CPP, and despite displaying symptoms, the cancer is not discovered until the patient interacts with the healthcare system in relation to other comorbidities or presents urgently with severe symptoms. This argument is substantiated by studies reporting that the suspicion of cancer is lowest among patients with a high customary use of primary care, and that these patients often have multiple morbidities [40]. However, having another disease may also bring the cancer forward at an earlier point in time – potentially even before the cancer symptoms become apparent. For instance, clinicians may discover hepatocellular carcinoma when surveilling patients with cirrhosis [44].

Recently more emphasis has been put on frailty rather than comorbidity to explain why older patients seem to be disadvantaged in healthcare, as two persons with same level of comorbidity may have substantially different level of health [45]. Frailty may be defined as an increased vulnerability and risk of adverse effects caused by reduced organ reserve capacity in a person, and is often measured by geriatrics using a comprehensive geriatric assessment (CGA) tool [45]. Using CGA have shown potential to improve prognosis in older patients with cancer [46], why a broader use of CGA (e.g. in primary care) may be useful. However, as current comprehensive geriatric assessment needs to be undertaken by an interdisciplinary team [46], a simpler and easier to use screening tool that could identify the patients most in need of a full CGA at a hospital could be useful.

While comorbidities and frailty are probable contributors to the age discrepancy in RtD, it is also possible that older patients are more likely to decline urgent referrals. Indeed, research suggests that while most old patients with cancer accept treatment, these patients are more likely to refuse invasive treatment compared to younger patients [47, 48]. Yet, we cannot rule out that some of the age discrepancy in RtD may also reflect a lower inclination among clinicians to offer speedy diagnostics to older patients with potential cancer.

## Conclusion

This study demonstrated that the route to diagnosis for patients with cancer varied by patients’ age. Patients with cancer aged 80 years or more were more likely to get diagnosed through unplanned admission (emergency), and both the younger patients (< 50 years) and the older patients (> 80 years) were less likely to be diagnosed after CPP referral from primary care compared to middle aged patients. These findings indicate that age disparities exist in relation to how patients are diagnosed with cancer in Denmark. Emphasis should be put on raising awareness among clinicians of cancer being the underlying cause of symptoms in both younger patients and in the older and often more comorbid patients. New diagnostic tools and pathways considering older patients needs and wishes should be developed.

## Supplementary Information

**Additional file 1.** Additional file - Age and RtD in DK 20220614.pdf.

## Data Availability

The datasets generated and analysed during the current study are not publicly available, as the data are stored and maintained electronically at Statistics Denmark, where it only can be accessed by pre-approved researchers using a secure VPN remote access due to the Danish legislation on data privacy. Furthermore, no data at a personal level are allowed to be subtracted from Statistics Denmark. Thus, the data in this project cannot be shared.

## Abbreviations

RtD: Routes to Diagnosis
CPP: Cancer Patient Pathway
GP: General Practitioner
ICD-10: International Classification of Diseases – version 10
DCO: Death Certificate Only
CCI: Charlson’s Comorbidity Index
ISCED: International Standard Classification of Education
CI: 95% Confidence Interval
CGA: Comprehensive Geriatric Assessment
