# Supplementary material for: Assessing how routes to diagnosis vary by the age of patients with cancer: a nationwide register-based cohort study in Denmark
Source: BMC Cancer. 2022 Aug 19;22:906. doi: 10.1186/s12885-022-09937-y (PMC9392355; doi:10.1186/s12885-022-09937-y)
Supplement: Supplementary file 1 — Additional file 1. Additional file - Age and RtD in DK 20220614.pdf. [file 12885_2022_9937_MOESM1_ESM.pdf]

Supplementary material for

Assessing how routes to diagnosis vary by the age of patients with cancer: A nationwide register-based cohort study in Denmark

1. National Screening programmes in Denmark

Table S1: Schematic overview of the national screening programmes for cancer in Denmark.

Cancer type	Sex & age group	Frequency	Test	Follow-up	Participation rate	Implemented since
Breast	Women 50-69 years	Biannual	Mammography	Clinical mammography, biopsy and clinical examination	83.7% (2019-2020)	2009
Cervical cancer	Women 23-64 years	Every three years	Smear-test & self-test for oldest age group	New smear or operation depending on the severity of findings	61.0% (2019)	Mid 1960's
Colorectal cancer	Men, women 50-74 years	Biannual	iFOBT cut-off value 100µg/L	Colonoscopy	60.2% (2020)	March 2014

Data source: Annual quality assurance reports (In Danish) from the Danish Clinical Registries – RKKP homepage (https://www.rkkp.dk/kvalitetsdatabaser/) accessed on 26 May 2022.

2. Definitions of group of diagnosis

We categorized cancer diagnoses into 23 specific groups of diagnosis based on the ICD-10 diagnosis code. The categorization was based on the Danish version of the "International Statistical Classification of Diseases and Health Related problems" set out by the WHO. Table S1 below outlines the categorization.

Table S2: Groups of cancer diagnosis, and the ICD-10 codes used to categorise the diagnosis within each group.

Diagnosis Group	ICD-10 codes
**Head & neck**	C000-C009; C019; C020-C024; C028-C031; C039-C041; C048-C052; C058-C062; C068-C069; C079-C081; C088-C092; C098-C104; C108-C113; C118; C119, C129-C132; C138-C140; C148
**Oesophagus**	C150-C159
**Stomach**	C160-C169
**Colon**	C180-C189
**Rectum**	C200-C209
**Liver**	C220-C229
**Pancreas**	C250-C259
**Lung**	C340-C349
**Malignant Melanoma**	C430-C439
**Breast (female)**	C500-C509
**Uterus**	C540-C549; C559
**Ovary**	C560-C569; C570-C579; C481-C489
**Female Genitals**	C510-C512; C518-C519; C529-C533; C574-C574; C577-C579
**Prostate**	C619
**Male genitals**	C600-C602; C608-C609; C620; C621; C629-C632; C637-C639
**Kidney**	C649; C659
**Bladder**	C670-C679
**Eye, Brain & CNS**	C690-C699; C700; C701; C709; C710-C719; C720-C729
**Endocrine glands**	C739; C740; C741; C749; C750-C759
**Lymphoma**	C810-C813; C817; C819; C820-C822; C827; C829; C830-C839; C840-C845; C850; C851; C857; C859
**Multiple myeloma**	C900-C903
**Leukaemia**	C892; C921-C929; C930-C932; C937; C939; C940-C945; C947; C950-C952; C957; C959
**Other**	All remaining ICD-10 codes

3. Patient demographics by routes to diagnosis

Table S3: Characteristics of the included patients with cancer stratified by routes to diagnosis (RtD) and total.

	Death certificate only	Screening	Cancer patient pathway, referral from primary sector	Cancer patient pathway, referral from secondary sector	Unplanned admission	Planned admission	Outpatient visit	Unknown route	Total
	n (%)	n (%)	n (%)	n (%)	n (%)	n (%)	n (%)	n (%)	n (%)
**Total**	630 (0.4)	9932 (.9)	66259 (46.2)	28843 (20.1)	22827 (5.9)	1495 (1.0)	8989 (6.3)	4414 (3.1)	143389 (100.0)
**Sex**									
Women	318 (50.5)	7597 (76.5)	30032 (45.3)	15192 (52.7)	10491 (46.0)	681 (45.6)	3560 (39.6)	2017 (45.7)	69888 (48.7)
Men	312 (49.5)	2335 (23.5)	36227 (54.7)	13651 (47.3)	12336 (54.0)	814 (54.4)	5429 (60.4)	2397 (54.3)	73501 (51.3)
**Age**									
mean, standard deviation	79.6 (10.3)	61.3 ((8.7)	66.8 (3.1)	66.6 (3.2)	71.0 (12.4)	65.1 (14.9)	67.2 (14.0)	68.2 (15.0)	67.2 (3.1)
**Age groups (years)**									
18-39		288 (2.9)	2566 (3.9)	1089 (3.8)	469 (2.1)	100 (6.7)	415 (4.6)	226 (5.1)	5153 (3.6)
40-49		211 (2.1)	4388 (6.6)	2256 (7.8)	766 (3.4)	142 (9.5)	573 (6.4)	272 (6.2)	8608 (6.0)
50-59	23 (3.7)	2983 (30.0)	9262 (14.0)	4114 (14.3)	2344 (10.3)	202 (13.5)	1236 (13.8)	549 (12.4)	20713 (14.4)
60-69	95 (15.1)	5151 (51.9)	19072 (28.8)	7914 (27.4)	5465 (23.9)	387 (25.9)	2394 (26.6)	1088 (24.6)	41566 (29.0)
70-79	164 (26.0)	1299 (13.1)	21028 (31.7)	9202 (31.9)	7872 (34.5)	445 (29.8)	2771 (30.8)	1256 (28.5)	44037 (30.7)
80-89	229 (36.3)		8880 (13.4)	3868 (13.4)	5060 (22.2)	185 (12.4)	1352 (15.0)	827 (18.7)	20401 (14.2)
90 or more	119 (18.9)		1063 (1.6)	400 (1.4)	851 (3.7)	34 (2.3)	248 (2.8)	196 (4.4)	2911 (2.0)
**Year of diagnosis**									
2014	140 (22.2)	2138 (21.5)	16679 (25.2)	6854 (23.8)	5773 (25.3)	404 (27.0)	2433 (27.1)	1161 (26.3)	35582 (24.8)
2015	168 (26.7)	2455 (24.7)	16381 (24.7)	7421 (25.7)	5744 (25.2)	342 (22.9)	2259 (25.1)	1052 (23.8)	35822 (25.0)
2016	134 (21.3)	2568 (25.9)	16560 (25.0)	7208 (25.0)	5690 (24.9)	351 (23.5)	2229 (24.8)	1117 (25.3)	35857 (25.0)
2017	188 (29.8)	2771 (27.9)	16639 (25.1)	7360 (25.5)	5620 (24.6)	398 (26.6)	2068 (23.0)	1084 (24.6)	36128 (25.2)
**Comorbidity (Charlson Comorbidity Index (CCI))**									
None	93 (14.8)	7177 (72.3)	38453 (58.0)	13710 (47.5)	8105 (35.5)	636 (42.5)	4812 (53.5)	2222 (50.3)	75208 (52.5)
Moderate (CCI=1-2)	170 (27.0)	2168 (21.8)	19649 (29.7)	9523 (33.0)	8424 (36.9)	517 (34.6)	2704 (30.1)	1424 (32.3)	44579 (31.1)
High (CCI>=3)	367 (58.3)	587 (5.9)	8157 (12.3)	5610 (19.5)	6298 (27.6)	342 (22.9)	1473 (16.4)	768 (17.4)	23602 (16.5)
**Educational level**									
Low	328 (52.1)	2519 (25.4)	21045 (31.8)	10026 (34.8)	9597 (42.0)	478 (32.0)	2842 (31.6)	1475 (33.4)	48310 (33.7)
Medium	196 (31.1)	4409 (44.4)	27602 (41.7)	11613 (40.3)	8644 (37.9)	576 (38.5)	3626 (40.3)	1789 (40.5)	58455 (40.8)
High	66 (10.5)	2879 (29.0)	16200 (24.4)	6501 (22.5)	3868 (16.9)	386 (25.8)	2281 (25.4)	1012 (22.9)	33193 (23.1)
Missing	40 (6.3)	125 (1.3)	1412 (2.1)	703 (2.4)	718 (3.1)	55 (3.7)	240 (2.7)	138 (3.1)	3431 (2.4)
**Disposable income (quantiles)**									
1st	170 (27.0)	1950 (19.6)	15744 (23.8)	7492 (26.0)	6304 (27.6)	351 (23.5)	2108 (23.5)	1023 (23.2)	35142 (24.5)
2nd	292 (46.3)	2017 (20.3)	16267 (24.6)	7605 (26.4)	7354 (32.2)	367 (24.5)	2211 (24.6)	1184 (26.8)	37297 (26.0)
3rd	127 (20.2)	2781 (28.0)	16213 (24.5)	6998 (24.3)	5368 (23.5)	362 (24.2)	2181 (24.3)	1020 (23.1)	35050 (24.4)
4th	41 (6.5)	3184 (32.1)	18035 (27.2)	6748 (23.4)	3801 (16.7)	415 (27.8)	2489 (27.7)	1187 (26.9)	35900 (25.0)
**Marital status**									
Single	426 (67.6)	2709 (27.3)	22951 (34.6)	10772 (37.3)	10774 (47.2)	549 (36.7)	3160 (35.2)	1847 (41.8)	53188 (37.1)
Married/co-habiting	204 (32.4)	7223 (72.7)	43308 (65.4)	18071 (62.7)	12053 (52.8)	946 (63.3)	5829 (64.8)	2567 (58.2)	90201 (62.9)
**Region**									
Northern Denmark	90 (14.3)	1120 (11.3)	6705 (10.1)	3057 (10.6)	2658 (11.6)	118 (7.9)	1320 (14.7)	485 (11.0)	15553 (10.8)
Central Denmark	135 (21.4)	2169 (21.8)	14816 (22.4)	6596 (22.9)	4982 (21.8)	245 (16.4)	1510 (16.8)	703 (15.9)	31156 (21.7)
Southern Denmark	125 (19.8)	2315 (23.3)	15169 (22.9)	6943 (24.1)	4990 (21.9)	332 (22.2)	2319 (25.8)	744 (16.9)	32937 (23.0)
Capital	175 (27.8)	2663 (26.8)	18020 (27.2)	7407 (25.7)	6658 (29.2)	545 (36.5)	2597 (28.9)	1781 (40.3)	39846 (27.8)
Zealand	105 (16.7)	1665 (16.8)	11549 (17.4)	4840 (16.8)	3539 (15.5)	255 (17.1)	1243 (13.8)	701 (15.9)	23897 (16.7)

Note: percentages may not total up to 100% due to rounding.

4. Multinominal regression results – symptomatic patients (i.e. all RtD excluding screening)

Table S4: underlying multinominal regression results for figure 1.

**RtD** variable		**Model A** n=133457		**Model B** n=133457		**Model C** n=133457	
		Relative risk ratio	(95%CI)	Relative risk ratio	(95%CI)	Relative risk ratio	(95%CI)
**DCO**							
sex	Woman	1	(ref)	1	(ref)	1	(ref)
	Man	0,93	(0,78-1,11)	0,77	(0,65-0,93)	1	(0,85-1,24)
Age group (years)	*18-39*	0	(0-0)	0	(0-0)	0	(0-0)
	40-49	0	(0-0)	0	(0-0)	0	(0-0)
	50-59	0,58	(0,37-0,92)	0,71	(0,45-1,12)	0,71	(0,45-1,12)
	60-69	1,00	(ref)	1,00	(ref)	1,00	(ref)
	70-79	1,56	(1,21-2,01)	1,31	(1,02-1,7)	1,19	(0,92-1,54)
	80-89	5,68	(4,45-7,25)	4,54	(3,55-5,8)	3,59	(2,8-4,61)
	90 or more	33,48	(25,09-44,66)	30,46	(22,73-40,83)	19,13	(14,08-26)
**CPP_primary_sector_referral**							
sex	Woman	1	(ref)	1	(ref)	1	(ref)
	Man	1	(ref)	1	(ref)	1	(ref)
Age group (years)	*18-39*	1	(ref)	1	(ref)	1	(ref)
	40-49	1	(ref)	1	(ref)	1	(ref)
	50-59	1	(ref)	1	(ref)	1	(ref)
	60-69	1	(ref)	1	(ref)	1	(ref)
	70-79	1	(ref)	1	(ref)	1	(ref)
	80-89	1	(ref)	1	(ref)	1	(ref)
	90 or more	1	(ref)	1	(ref)	1	(ref)
**CPP__secondary_sector_referral**							
sex	Woman	1	(ref)	1	(ref)	1	(ref)
	Man	0,92	(0,89-0,95)	0,90	(0,86-0,93)	0,90	(0,87-0,94)
Age group (years)	*18-39*	1,23	(1,13-1,34)	1,38	(1,26-1,5)	1,37	(1,26-1,49)
	40-49	1,31	(1,23-1,39)	1,43	(1,35-1,53)	1,45	(1,36-1,54)
	50-59	1,06	(1,01-1,11)	1,11	(1,06-1,17)	1,12	(1,07-1,18)
	60-69	1	(ref)	1,00	(ref)	1,00	(ref)
	70-79	1,04	(1-1,08)	0,99	(0,96-1,03)	0,99	(0,95-1,02)
	80-89	1,03	(0,98-1,08)	0,95	(0,91-1)	0,94	(0,9-0,99)
	90 or more	0,96	(0,85-1,08)	0,90	(0,79-1,01)	0,87	(0,77-0,99)
**Unplanned_admission**							
sex	Woman	1	(ref)	1	(ref)	1	(ref)
	Man	0,99	(0,95-1,02)	0,94	(0,91-0,98)	1,03	(1-1,07)
Age group (years)	*18-39*	1,10	(0,98-1,23)	1,36	(1,21-1,52)	1,32	(1,18-1,49)
	40-49	0,98	(0,9-1,07)	1,15	(1,05-1,26)	1,22	(1,11-0,33)
	50-59	0,97	(0,92-1,03)	1,06	(1-1,12)	1,08	(1,02-1,14)
	60-69	1	(ref)	1,00	(ref)	1,00	(ref)
	70-79	1,29	(1,23-1,34)	1,19	(1,14-1,24)	1,13	(1,09-1,18)
	80-89	1,97	(1,88-2,07)	1,75	(1,67-1,84)	1,56	(1,48-1,64)
	90 or more	3,31	(2,98-3,66)	3,01	(2,71-3,33)	2,42	(2,17-2,69)
**Planned_admission**							
sex	Woman	1	(ref)	1	(ref)	1	(ref)
	Man	0,91	(0,8-1,03)	0,87	(0,77-0,99)	0,88	(0,77-1)
Age group (years)	*18-39*	2,21	(1,73-2,83)	2,70	(2,1-3,47)	2,64	(2,05-3,4)
	40-49	2,00	(1,63-0,47)	2,33	(1,89-2,87)	2,28	(1,85-2,82)
	50-59	1,10	(0,93-1,31)	1,19	(1-1,42)	1,18	(0,98-1,4)
	60-69	1	(ref)	1,00	(ref)	1,00	(ref)
	70-79	1,08	(0,94-1,24)	1,00	(0,87-0,15)	1,00	(0,87-1,15)
	80-89	1,08	(0,94-1,24)	0,96	(0,8-1,15)	0,96	(0,8-1,15)
	90 or more	1,79	(0,94-1,24)	1,61	(1,13-2,31)	1,50	(1,04-2,16)
**Outpatient**							
sex	Woman	1	(ref)	1	(ref)	1	(ref)
	Man	0,92	(0,87-0,97)	0,90	(0,85-0,96)	0,91	(0,86-0,97)
Age group (years)	*18-39*	1,53	(1,36-1,73)	1,61	(1,42-1,82)	1,60	(1,41-1,81)
	40-49	1,40	(1,26-1,55)	1,46	(1,31-1,61)	1,45	(1,3-1,61)
	50-59	1,16	(1,08-1,25)	1,19	(1,1-1,28)	1,19	(1,1-1,28)
	60-69	1	(ref)	1,00	(ref)	1,00	(ref)
	70-79	1,07	(1,01-0,13)	1,04	(0,98-1,1)	1,04	(0,98-0,11)
	80-89	1,25	(1,16-0,35)	1,20	(1,12-1,3)	1,20	(1,11-1,29)
	90 or more	2,05	(1,77-2,38)	1,99	(1,71-2,3)	1,92	(1,65-2,24)
**Unknown**							
sex	Woman	1	(ref)	1	(ref)	1	(ref)
	Man	0,83	(0,77-0,89)	0,82	(0,76-0,88)	0,84	(0,78-0,9)
Age group (years)	*18-39*	1,52	(1,29-1,78)	1,63	(1,39-1,92)	1,63	(1,38-1,92)
	40-49	1,27	(1,1-1,47)	1,35	(1,17-1,55)	1,36	(1,17-0,57)
	50-59	1,08	(0,97-1,2)	1,12	(1-0,25)	1,11	(1-0,24)
	60-69	1	(ref)	1,00	(ref)	1,00	(ref)
	70-79	1,05	(0,97-1,14)	1,02	(0,93-1,11)	1,00	(0,92-1,09)
	80-89	1,66	(1,51-1,83)	1,58	(1,43-1,73)	1,50	(1,36-1,65)
	90 or more	3,40	(2,87-4,02)	3,23	(2,73-3,83)	2,85	(2,39-3,39)

Model A: adjusted for sex, diagnosis group, region of residence, and year of diagnosis.
Model B: adjusted for sex, diagnosis group, region of residence, year of diagnosis, and comorbidities.
Model C: Adjusted for sex, diagnosis group, region of residence, year of diagnosis, comorbidities, immigration status, cohabitation status, income, and educational level.

5. Marginsplot for the case mix adjusted model (Model A -Table S3) and for the model that is case mix adjusted and adjusted for comorbidities (Model B -Table S3)

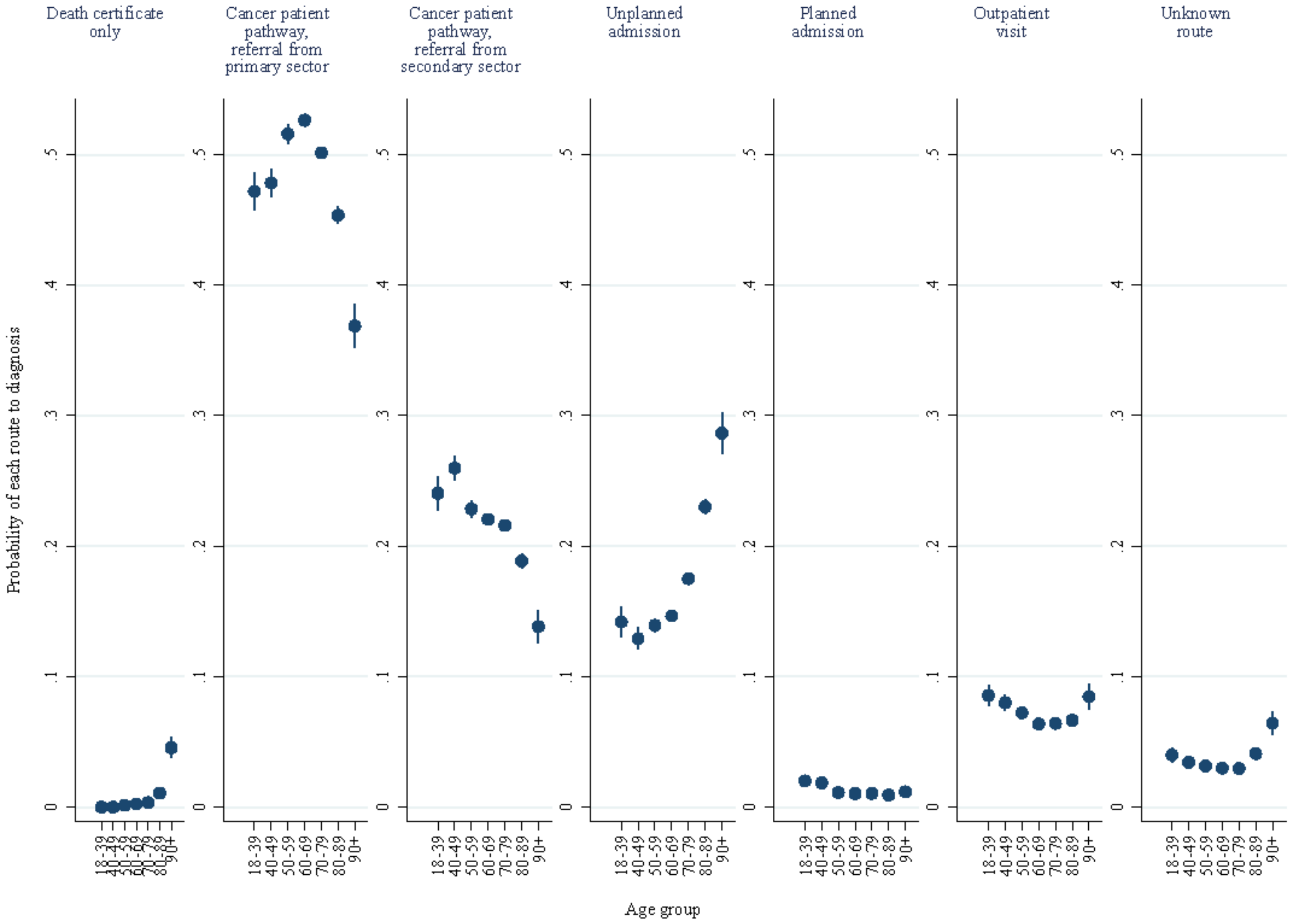

Figure S1: Marginal probability of a cancer diagnosis via each route to diagnosis by age groups based on the case-mix adjusted multinomial regression model (covariates included sex, diagnosis group, region of residence, and year of diagnosis). Patients diagnosed through the screening route were excluded.

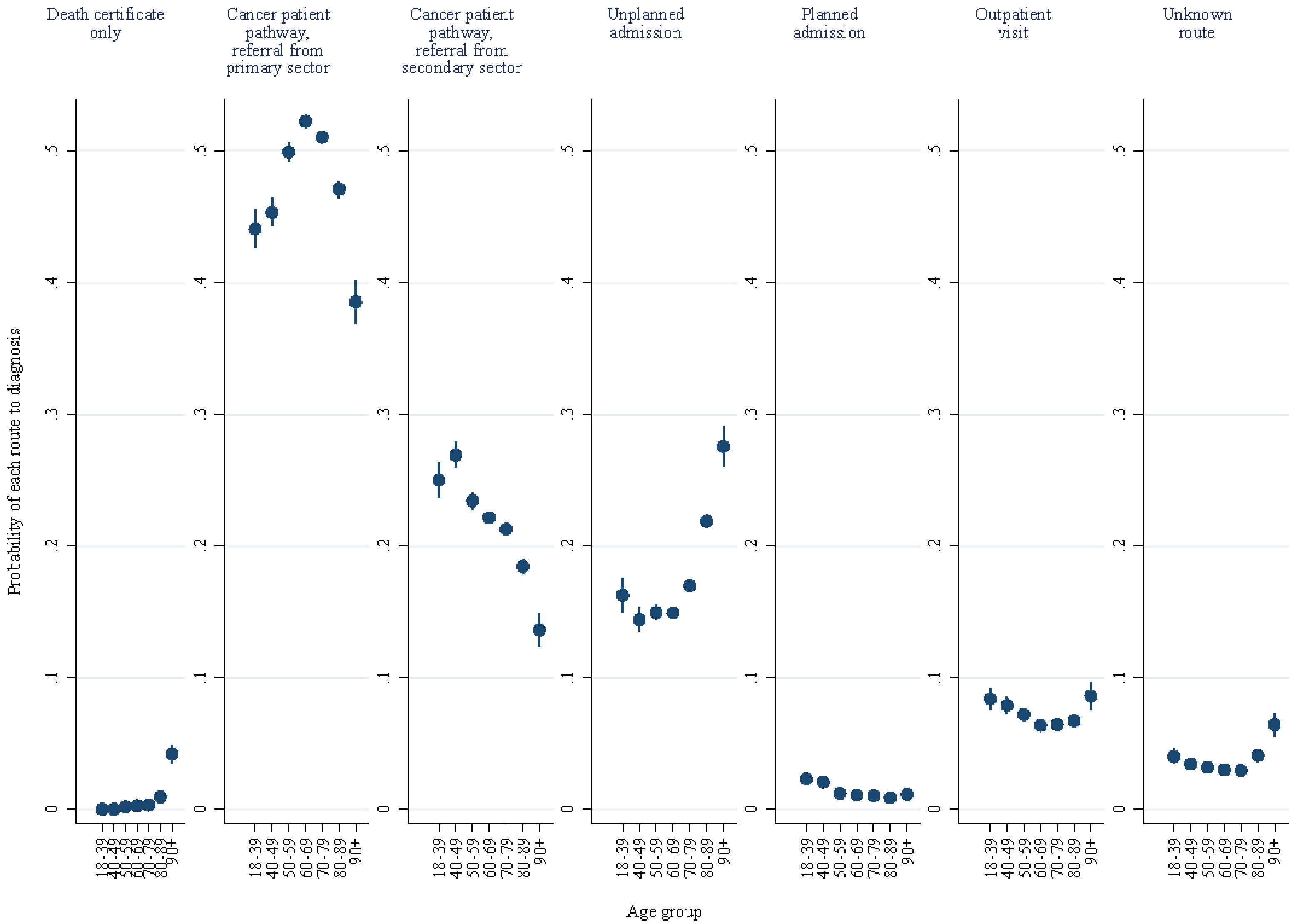

Figure S2: Marginal probability of a cancer diagnosis via each route to diagnosis by age groups based on the case-mix and comorbidity adjusted multinomial regression model (covariates included sex, diagnosis group, region of residence, year of diagnosis, and comorbidities). Patients diagnosed through the screening route were excluded.

6. Analyses investigating RtD by age groups across the four main cancer types (breast, colon, lung, and prostate).

To facilitate the analyses and avoid small cells, the two routes DCO and planned admission were excluded. DCO constituted 0.2 – 1.1 percent of the four cancers, while planned admission constituted 0.2 – 1-2 percent. In addition, as this analyses focused on the symptomatic cancers, screening was excluded – which denoted 28.2 percent of breast cancer cases and 18.2 of colon cancer cases. To further accommodate issues related to small cells, cells with fewer than 10 cases were set to 0.
The analyses were performed as multinomial regression model (adjusted for sex, year of diagnosis, region, comorbidities, education, income, marital status, and immigration status) separately for each of the four cancer sites (similar model as for the main analyses).

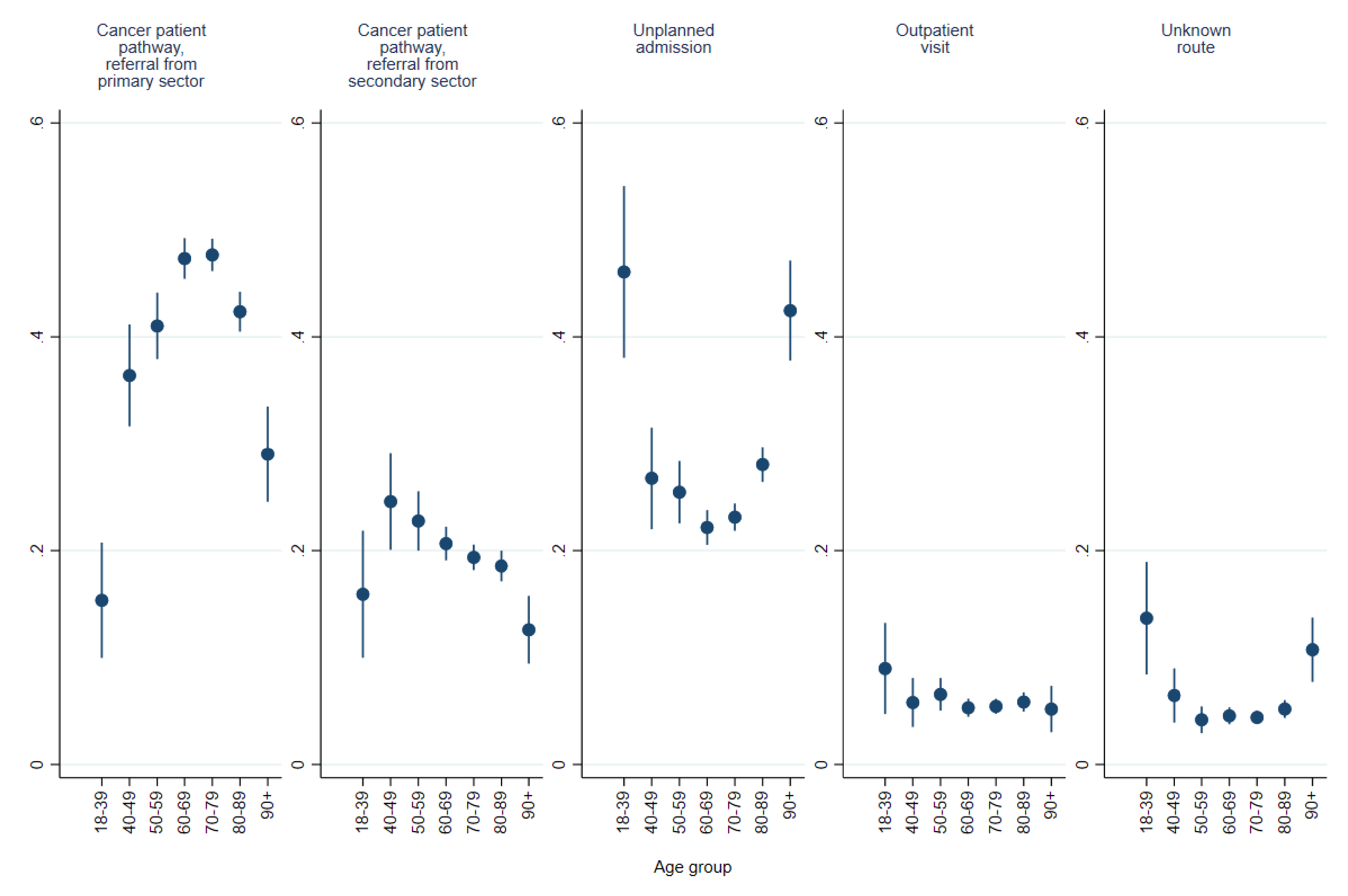

Figure S3: Marginal probability of a ***colon cancer*** *diagnosis via each route to diagnosis by age groups based on the fully adjusted multinomial regression model (covariates include sex, year of diagnosis, region, comorbidities, education, income, marital status, and immigration status). Patients diagnosed through the screening route, DCO and planned admission were excluded.*

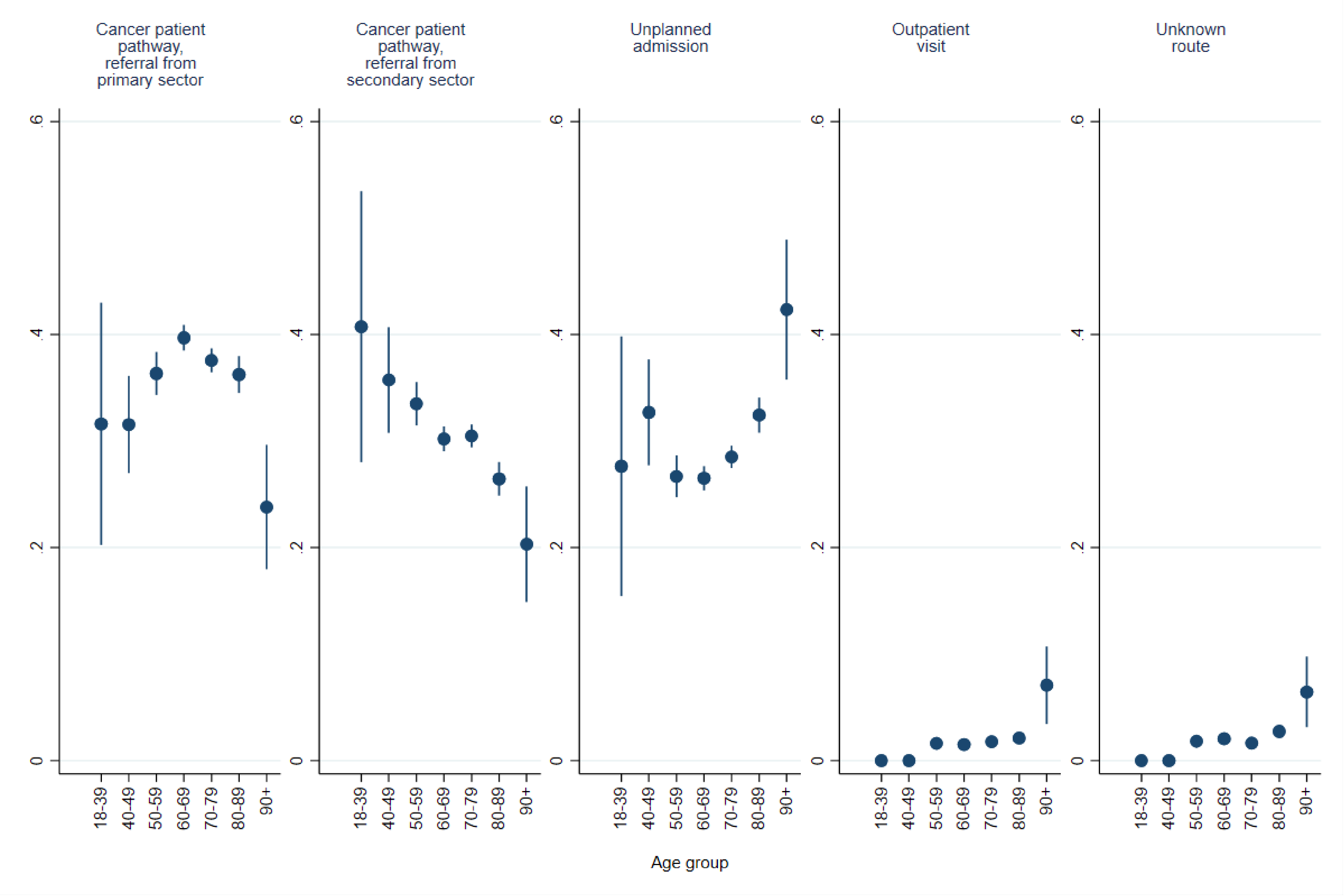

Figure S4: Marginal probability of a **lung cancer diagnosis** via each route to diagnosis by age groups based on the fully adjusted multinomial regression model (covariates include sex, year of diagnosis, region, comorbidities, education, income, marital status, and immigration status). Patients diagnosed through the screening route, DCO and planned admission were excluded.

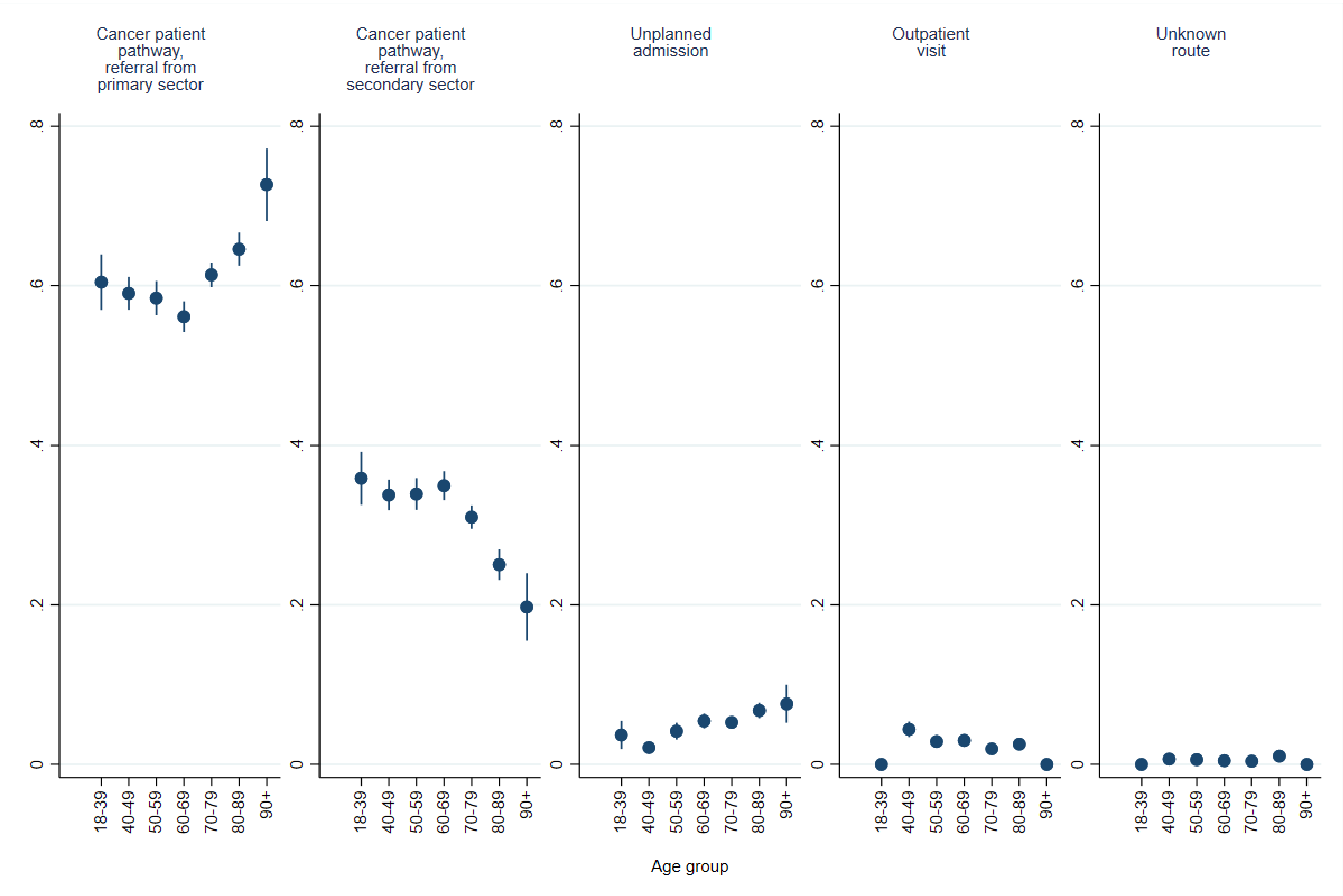

Figure S5: Marginal probability of a **breast cancer diagnosis** via each route to diagnosis by age groups based on the fully adjusted multinomial regression model (covariates include sex, year of diagnosis, region, comorbidities, education, income, marital status, and immigration status). Patients diagnosed through the screening route, DCO and planned admission were excluded.

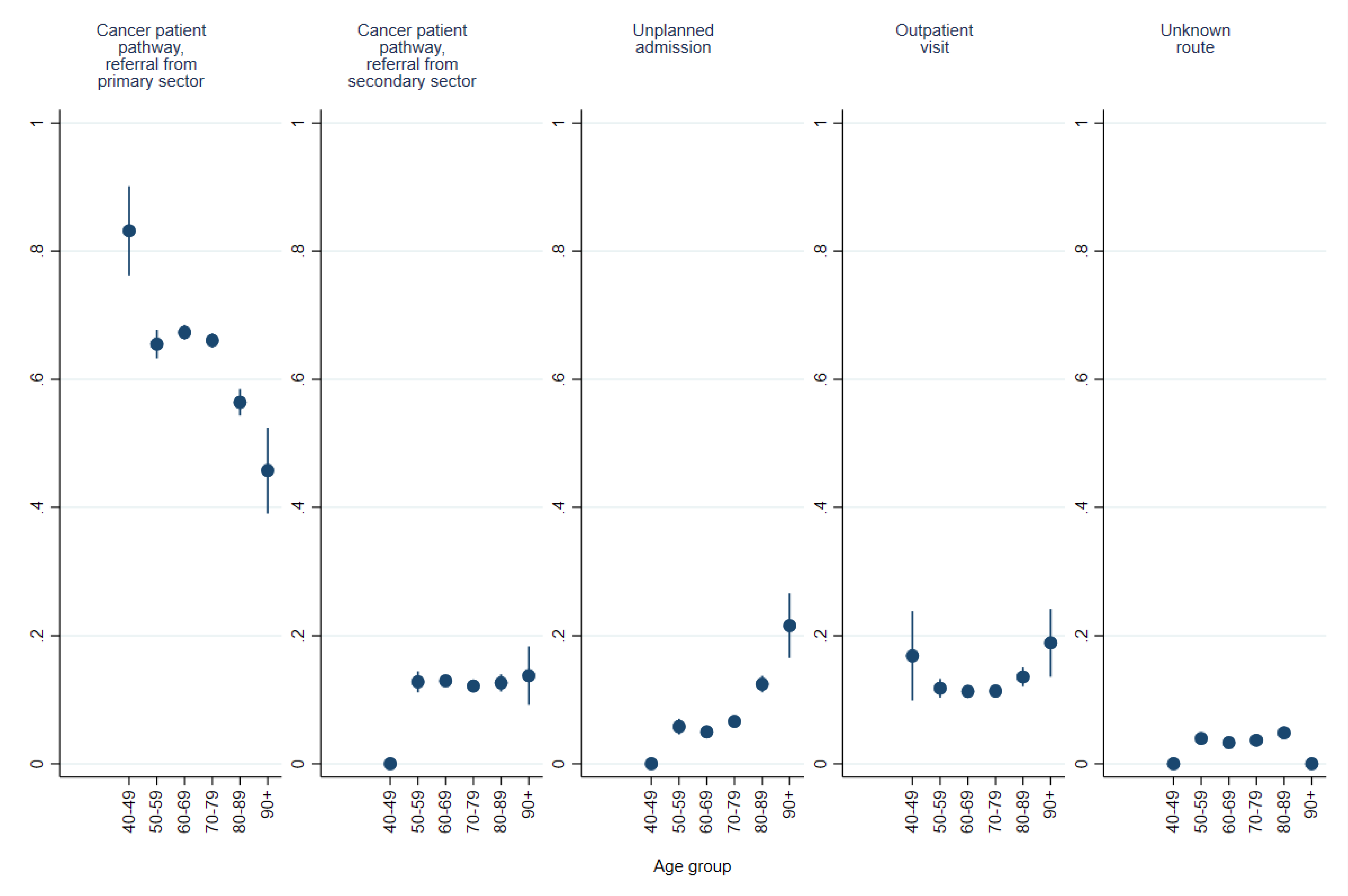

Figure S6: Marginal probability of a **prostate cancer diagnosis** via each route to diagnosis by age groups based on the fully adjusted multinomial regression model (covariates include sex, year of diagnosis, region, comorbidities, education, income, marital status, and immigration status). Patients diagnosed through the screening route, DCO and planned admission were excluded.

7. Marginsplot after exclusion of diagnosis with an age restriction in CPP guidelines

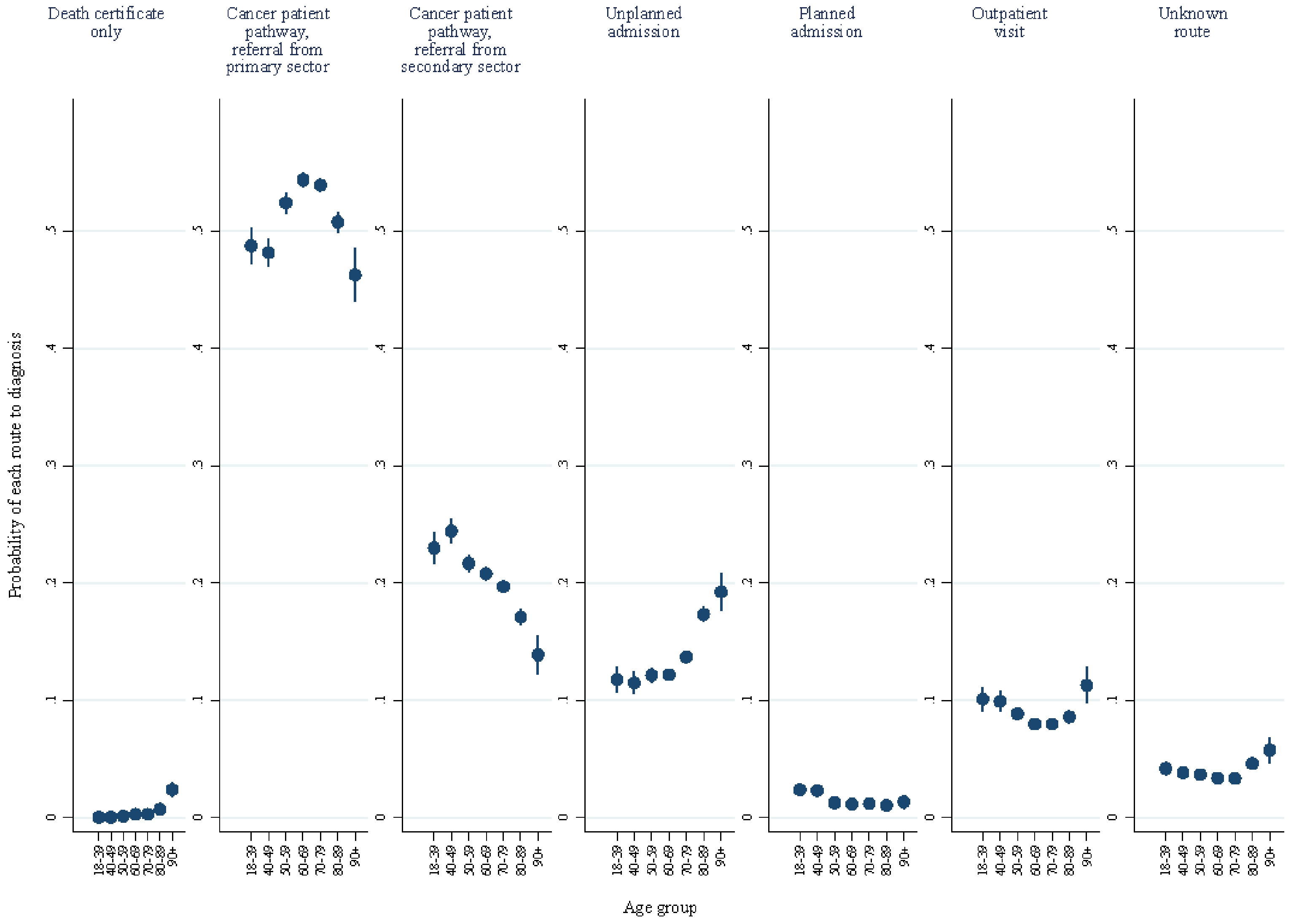

Figure S7: Marginal probability of each routes to diagnosis by age groups, after exclusion of diagnosis with an age restriction in the Cancer Patient Pathways (CPPs) (covariates included sex, diagnosis group, region of residence, year of diagnosis, and comorbidities). Patients diagnosed through the screening route were excluded.

8. Marginsplot regarding whether the association between age and RtD depended on patients' level of comorbidities

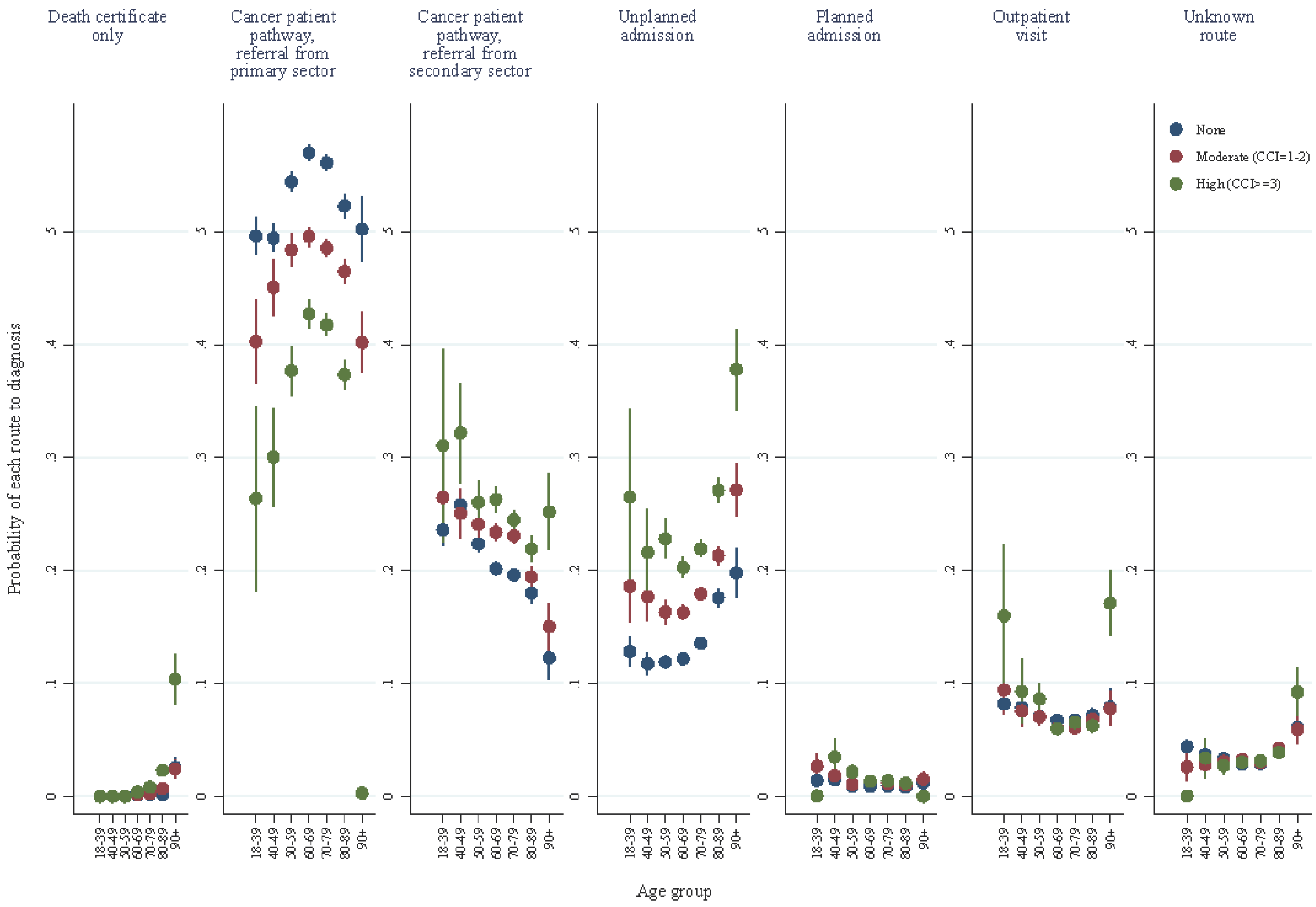

Note: Groups with 10 observations or less were set to 0 observations

Figure S8: Marginal probability of a cancer diagnosis via each route to diagnosis by age groups and level of comorbidities (covariates included sex, year of diagnosis, region, diagnosis group, comorbidities, education, income, marital status, and immigration status). Patients diagnosed through the screening route were excluded. Note: Groups with 10 observations or fewer were set to 0 observations.

9. Logistic regression results regarding whether patients are diagnosed via the screening route

Table S5: underlying logistic regression results for figure 3.

		Colon				Rectum				Breast				Cervical cancer			
**RtD**		**Model A** n=11019		**Model B** n=11019		**Model A** n=5330		**Model B** n=5330		**Model A** n=9698		**Model B** n=9698		**Model A** n=1374		**Model B** n=1374	
		OR	(95%CI)	OR	(95%CI)	OR	(95%CI)	OR	(95%CI)	OR	(95%CI)	OR	(95%CI)	OR	(95%CI)	OR	(95%CI)
sex	Woman	1.00		1.00		1.00		1.00									
	Man	1.20	(1.1-1.3)	1.09	(1-1.19)	1.31	(1.14-1.5)	1.20	(1.04-1.38)								
Age group (years)	*18-39*													2.17	(1.54-3.07)	1.72	(1,19-2,47)
	40-49													1.29	(0.82-2.04)	1.18	(0,74-1,88)
	50-59	0.95	(0.84-1.09)	0.91	(0.8-0.04)	0.84	(0.7-1)	0.83	(0.69-1)	0.75	(0.69-0.81)	0.74	(0.68-0.8)	0.77	(0.48-1.24)	0.74	(0,46-0,2)
	60-69	1.00		1.00		1.00		1.00		1.00		1.00		1.00		1.00	
	70-79	0.94	(0.85-1.04)	0.99	(0.89-1.1)	0.92	(0.78-1.08)	0.98	(0.83-1.15)								

Model A: adjusted for diagnosis group, region of residence, and year of diagnosis.
Model B: Adjusted for diagnosis group, region of residence, year of diagnosis, comorbidities, immigration status, cohabitation status, income, and educational level.

10. Marginsplot for case-mix adjusted logistic regression regarding whether patients are diagnosed via the screening route (Model A)

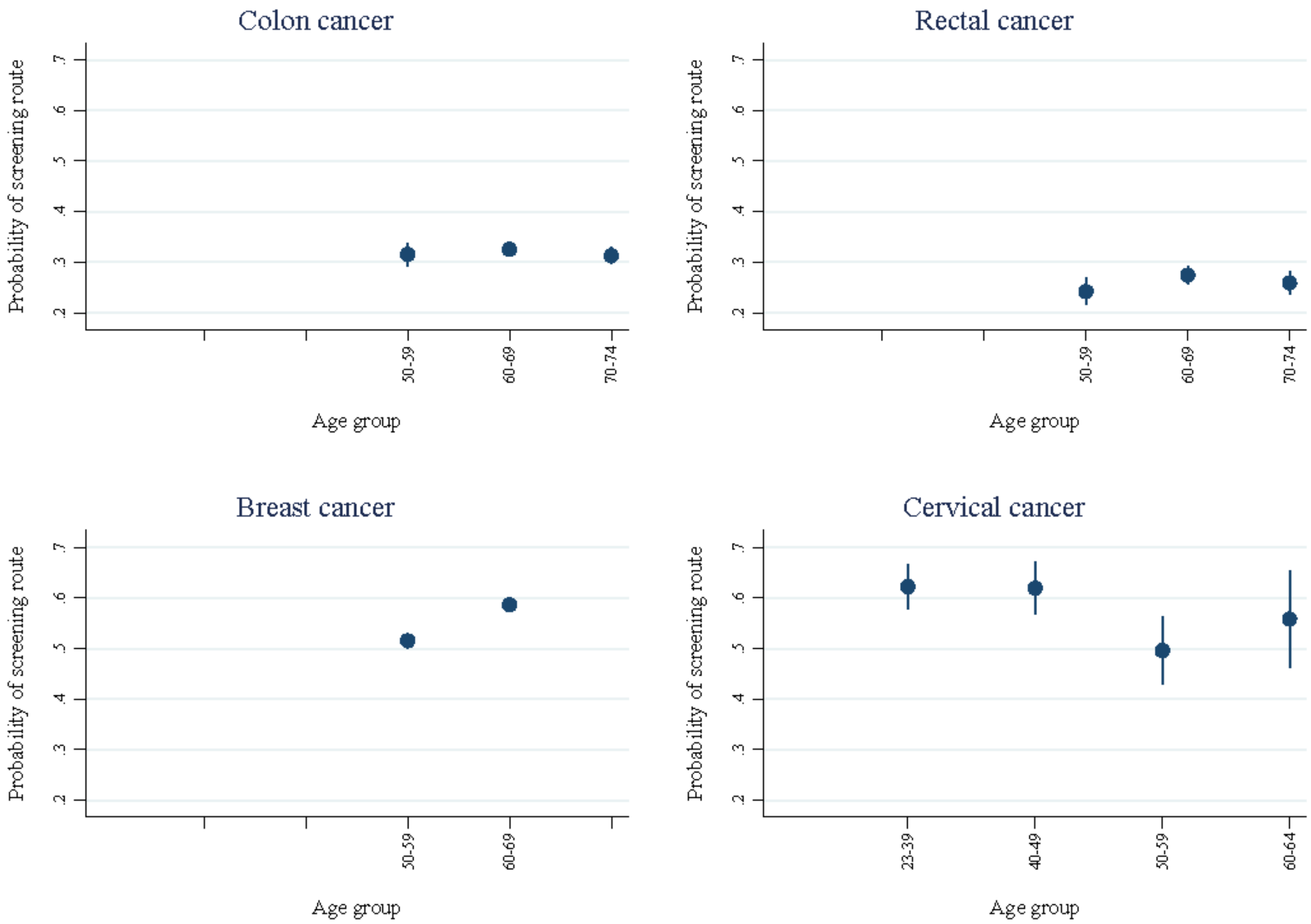

Figure S9: Marginal probability of screening by age groups for cancer sites with a national screening programme – case-mix adjusted model.
